# Two-layer dynamic blood phantom for assessing NIRS device accuracy

**DOI:** 10.1364/BOE.569310

**Published:** 2025-08-07

**Authors:** E. Russomanno, X. Yang, H. Zohdi, J. Jiang, M. Ackermann, L. Lanini, D. Yacheur, M. Wolf, A. Kalyanov

**Affiliations:** 1 Biomedical Optics Research Laboratory (BORL), Department of Neonatology, University Hospital Zurich, University of Zurich, Zurich, Switzerland; 2 D-ITET, ETH Zurich, Switzerland; 3 Institute of Complementary and Integrative Medicine, University of Bern, Bern, Switzerland

## Abstract

Near-infrared spectroscopy (NIRS) is a widely used non-invasive method for measuring oxygenation and hemodynamics. NIRS devices are validated using phantoms that accurately replicate the optical properties of human tissue. The aim was to develop a multi-layer blood-lipid phantom specifically to mimic the layered anatomical structure of the human head. The phantom consists of two adjustable liquid layers, which model the optical properties and dynamic characteristics of brain and superficial tissues, together with two fixed solid layers. We demonstrate our phantom capabilities by testing the INVOS 7100 and Masimo O3 NIRS devices. The phantom enabled the Masimo instrument to show that it is less influenced by superficial changes (RMSE relative to superficial layer ∼50%) than the INVOS instrument (∼35%), highlighting better depth sensitivity. This shows the phantom’s value as a tool for guiding device development and evaluation.

## 1. Introduction

Near-infrared spectroscopy (NIRS) represents a non-invasive optical technique that utilizes near-infrared light to investigate biological tissues. NIRS technology has become widely adopted across biomedical fields, because it provides information about tissue oxygenation and blood volume dynamics. In operating rooms and intensive care units, NIRS-based cerebral oximetry monitors brain oxygenation and identifies possible hypoxic or ischemic events [1,2]. Accurate validation methods are essential for reliable deep tissue oxygenation measurements of NIRS [3]. Validating NIRS technology requires tissue-simulating models called phantoms that mimic tissue-specific optical properties, including reduced scattering (μ_s_’) and absorption (μ_a_) coefficients. Phantoms create a controlled and standardized testing environment, which eliminates the ethical and methodological problems that arise from invasive validation methods. Invasive validation techniques are lacking a direct measure of oxygenation to compare NIRS to. Therefore, they require substitutes such as sampling arterial and venous blood form the jugular bulb blood during breathing of hypobaric oxygen concentrations. This requires several assumptions, e.g., about the compartment sizes of the venous and arterial compartments, the representativeness of the jugular bulb blood for the volume that is measured by NIRS etc. In addition, these invasive methods are not applicable in preterm infants for safety reasons [4,5]. In previous phantom tests, different brands of oximeters showed systematically different oxygenation values, which were linearly correlated. This highlights the benefit of standardized validation phantoms [4]. The difference between commercial NIRS oximeters results from different source-detector separations, wavelengths and algorithms to calculate tissue oxygenation. Phantom enabled to correlate the different instruments and generate conversion tables.

The development of tissue-simulating phantoms for NIRS has made significant progress over the last few decades. Previous phantoms e.g. did not simulate the layered structure of human tissues adequately. Studies with multi-layered phantoms showed that NIRS devices are influenced by the optical properties of the different layers [6–8]. Scattering is generated by lipid-based emulsions or polymer microspheres. The range of absorbers in NIRS studies extends from biological molecules such as hemoglobin to stable molecular compounds such as dyes and inks. Solid phantoms made of resin and silicone maintain optical properties over extended periods and are needed for static calibration and drift assessment. Solid phantoms cannot simulate biological tissue dynamics. Whereas in liquid phantom optical stability needs to be preserved by continuous mixing and accurate control of temperature and pH levels. Conventional liquid phantoms can adjust optical properties but do not effectively replicate anatomical structures.

One question about NIRS devices is how accurately they measure the oxygenation of deep tissue [9]. For this purpose, it is important to capture the multi-layer nature of biological tissues. Haeussinger et al. showed in simulations discrepancies in oxygen saturation (StO_2_) readings, when superficial and deep tissues are assessed collectively [3]. Dantuma et al., found that multi-layered phantoms are essential in simulating complex biological environments and that a single-layer structure cannot represent these sufficiently [10]. Earlier research by Kleiser et al. [4] conducted device comparisons using single-layer containers, whereas Lange et al. [11] conducted experiments by placing optical fibers directly into a single-layer liquid. The design by Del Bianco et al. [12] for multi-layer liquid phantoms included statically embedded mylar foils. Recently, Sudakou et al. [6] presented an excellent two-compartment phantom, which operates without solid layers and uses an external pump and movable internal container. The limitation here is that it cannot accommodate larger probes such as the Pioneer System [13]. Another limitation was the lack of an independent reference measurement of the true StO_2_ [6]. The aim was to develop and test a multi-layer blood-lipid phantom representing the human head with two liquid layers that are adjustable in optical properties and thickness, one representing skin/muscle and one the brain; and two solid layers, one of them simulating skull tissue. The phantom enables including independent reference measurements, precise optical adjustments and environmental control. A further aim was to test this phantom in vitro with two NIRS devices: the INVOS 7100 and Masimo O3.

## 2. Materials and methods

### 2.1. Tissue optics variability

To accurately model and validate NIRS requires μ_a_, μ_s_′, refractive index, and anisotropy factor to correspond to the ones of the human head [14]. In addition, the adult head consists of superficial layers such as scalp and skin, intermediate layers such as skull, and deeper layers including brain and cerebrospinal fluid. In superficial tissues, we considered an μ_a_ range between 0.05 and 0.15 cm^−1^ and μ_s_′ between 8 and 12 cm^−1^. For brain tissue, μ_a_ ≈ 0.10-0.20 cm^−1^ and μ_s_′ ≈ 6-14 cm^−1^ [15]. The skull thickness generally spans 5 to 10 mm while scalp thickness lies between 5 and 8 mm [16,17]. The cerebral total hemoglobin concentrations (tHb) vary between publications within the range of 26 µM to 104 µM [18–22]. There is a dependence on age: 52 ± 3 µM in young adults and 38 ± 2 µM in older adults [23]. A gender difference was observed with tHb values of 81 ± 15 µM in females and 104 ± 24 µM in males aged 16-18 years [21]. Others measured a tHb of 26.8 ± 3.9 µM during visual stimulation [19]. Mean values of approximately 70 ± 10 µM, were obtained using frequency-domain (FD) NIRS and a homogeneous model for data analysis [20]. The variability in tHb needs to be considered to achieve a reliable phantom-based validation. Based on these findings, the tHb in our phantom experiments was set between a minimum of 26 µM and a maximum of 75 µM.

### 2.2. Phantom design

**Fig. 1.**
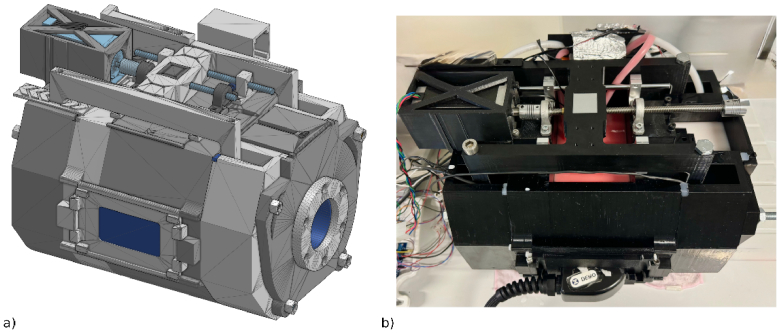
Dual-layer NIRS phantom design. a) CAD model of the complete phantom assembly, showing the dual-layer container structure with integrated mounts for optical sensors, flow channels, and mechanical components for fluid actuation. b) Photograph of the physical phantom during an experiment including tubing for fluid circulation, stepper-driven motor system, and wiring for sensor integration.

At BORL, we developed a sophisticated multi-layered phantom container (Fig. 1) to accurately replicate human tissue optical characteristics for NIRS measurements. This advanced phantom contains several features that enable to accurately simulate the layered structure of human head anatomy and biological conditions.

**Fig. 2.**
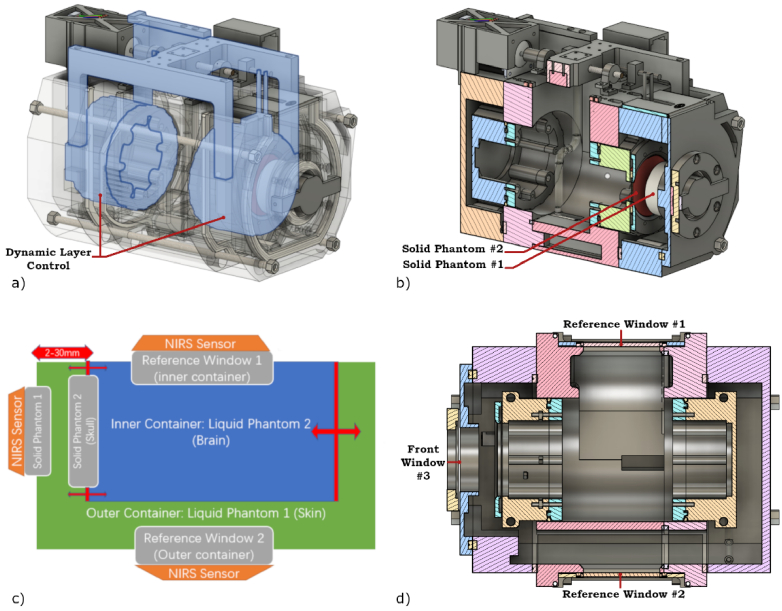
Structural and optical layout of the dual-container NIRS phantom. a) Transparent CAD rendering of the phantom assembly, illustrating the displacement mechanism (light blue) that adjusts the superficial layer thickness (SLT). b) Vertical cross-section rendering depicting the arrangement of the deep (brain) and superficial liquid compartments, along with sealing and actuation elements. c) Schematic top view of the horizontal cross-section, showing the positions of the deep and superficial layers and the reference and front windows. d) Horizontal cross-section rendering detailing the three optical interface windows: reference window #1 (deep layer), reference window #2 (superficial layer), and front window #3 for the test probe measuring the combined dual-layer volume.

The design integrates both deep and superficial compartments structured to accommodate solid and liquid phantoms, which may represent various tissue types. The container comprises four main sections, depicted in different shades of grey in the CAD rendering (Fig. 1(a)). It is 3D printed with chemically resistant and mechanically robust PETG material. The parts have been assembled into a solid frame by stainless steel screws (Fig. 2(a)). Silicone gaskets between sections deliver strong sealing capabilities that block leakage. The superficial liquid compartment represents extracerebral tissues such as skin and scalp, and its thickness can be automatically adjusted from 2 mm to 30 mm. The adjustment mechanism is powered by a high-torque NEMA 23 stepper motor, which connects through an M8 lead screw to linear guide rails and a linkage platform to provide smooth and precise positioning.

The inner container houses the liquid phantom simulating the brain, which is separated from the superficial liquid phantom by a changeable window of solid silicone phantom (labeled in Fig. 2(b)) representing the skull bone layer, cerebrospinal fluid, or both (Solid Phantom #2 in Fig. 2(b)). To adjust the thickness of the superficial liquid phantom, the front and back walls of the inner container are moved synchronously. The mechanism is illustrated in Fig. 2(a), where the moving components are highlighted in light blue. This synchronous displacement ensures a constant liquid level within the inner container, thereby preventing pressure differences that could deform the solid phantom (skull) or cause sensor displacement on the outer window. This mechanism allows precise control of the superficial layer thickness while ensuring stable and accurate NIRS measurements. The frontal window is ∼0.9 mm thick, and the internal window separating the superficial and brain layers is ∼1.9 mm thick. The solid phantom windows can be molded with custom optical properties and thicknesses. In this case, properties similar to the average liquid mixture were selected: μ_s_′ = ∼10 cm^-1^ and µa = ∼0.10 cm^−1^.

For the two reference windows (labeled in Fig. 2(c)-(d)), sized at 50 mm by 90 mm, we used the same thickness and optical properties as in [4], which allowed accurate, independent and simultaneous monitoring of the optical properties of both liquid layers. Behind each reference window, there is a volume of the respective liquid with a minimum thickness of 50 mm to ensure semi-infinite conditions and minimize crosstalk between compartments. The multilayer configuration enables realistic testing of devices through its primary window (Pos. #3 in Fig. 2(d)), which has a radius of 65 mm. Both homogeneity and temperature stability are tightly controlled by three internal pumps that circulate liquid at 1 liter/min rate within the liquid compartments to maintain constant optical properties.

To maintain physiological temperature stability (∼37°C), liquids circulate through three heating metals integrated in the pump. The phantom includes the following sophisticated sensor systems: i) an Arduino UNO microcontroller manages all mechanical and electronic elements in the system and enables automated control of the superficial layer thickness (SLT) by the stepper motor; ii) the GP2Y0A41 infrared distance sensor mounted on the movable linkage platform ensures accurate monitoring of the SLT values; iii) two sensors precisely control and monitor liquid levels in each compartment while sending real-time data to the microcontroller; iv) both temperature and pH levels are monitored with immersed temperature probes and pH sensors with an accuracy of ±0.1 pH units; and v) the phantom incorporates covers to minimize oxygen diffusion from the air and openings for additional sensors or adding reagents such as yeast, blood, and/or oxygen. The operation of the phantom was confirmed during testing. SLT errors were < 0.5 mm, and there was negligible leakage.

### 2.3. NIRS oximeters

The aim was to evaluate the performance of two commercial NIRS devices with probes for adults in the phantom, particularly their sensitivity for the deep layer. The Masimo O3 Regional Oximeter is CE marked for medical application and employs wavelengths at 690, 760, 805, and 880 nm to distinguish between oxyhemoglobin (O_2_Hb) and deoxyhemoglobin (HHb) and to determine regional StO_2_ [24]. The device uses disposable adhesive sensors with 30 mm and 40 mm source-detector separations (SDS). The INVOS 7100 Cerebral/Somatic Oximetry System measures CE marked for medical application and utilizes two wavelengths of 730 and 810 nm with SDS of 30 mm and 40 mm to measure StO_2_ continuously [25]. The OxiplexTS NIRS frequency-domain oximeter from ISS, Inc., CE marked for research, served as the reference for monitoring μ_a_, μ_s_′ and StO_2_. The device operates at a modulation frequency of 110 MHz at 692 nm and 834 nm wavelengths. The OxiplexTS probe features SDS of 25, 30, 35, and 40 mm, which were shown to accurately measure O_2_Hb, HHb and StO_2_ [26].

### 2.4. Protocol

For each experiment, both the inner and outer containers were filled with 4000 ml of phosphate-buffered saline (PBS) at pH 7.4, pre-heated to approximately ∼38°C to reflect physiological conditions. The optical probes were attached to their specific windows on the phantom container. Each probe type had its own custom-printed holder to ensure consistent placement and tight optical coupling during the experiments. Window #1 (Fig. 2(d)) hosted the OxyplexTS reference optode allocated to the deep layer. The OxyplexTS reference optode for the superficial layer was installed at window #2 (Fig. 2(d)). This enabled us to monitor the optical properties and StO_2_ of the phantom continuously. The NIRS probe under evaluation was placed on window #3. We then introduced the Smoflipid 20% (Fresenius Kabi) lipid emulsion to simulate tissue-like scattering properties. We gradually increased its amount to reach the targeted μ_s_′ = 9.8 cm^−1^, consistent with values reported for adult brain tissue in the literature [15]. We achieved the desired μ_a_ and tHb levels by adding human erythrocyte concentrate in increments from a blood bag, obtained from Blutspende Zürich.

During the experiments, dynamic StO_2_ changes were induced in one layer, while the other layer was maintained at 100% oxygen saturation. Deoxygenation: we started controlled deoxygenation by introducing active baker's yeast (Saccharomyces cerevisiae, 3 g) and a glucose solution (∼50%, 3 ml) to the liquid. The yeast's aerobic respiration caused StO_2_ to decrease. This process was quantitatively monitored by the OxiplexTS, which showed when a stable minimum was reached. Oxygenation: pure O_2_ gas was infused through a tube. This resulted in a quick hemoglobin re-saturation. pH Control: The metabolic process of yeast generates carbon dioxide, which may decrease pH levels. We administered small natrium bicarbonate solution aliquots (typically 15 ml) to maintain a physiological pH ≈ 7.3-7.4, which was monitored intermittently.

Protocols and scattering parameters (Table 1) were adjusted iteratively across experiments to reflect realistic variability in tissue properties and to optimize the measurement conditions. E.g. in Exp. 1 we observed large effects and therefore decided to add a shorter SLT of 6 mm. Since the reduced scattering coefficient varies between subjects, we also varied it intentionally within the range of expected values.

**Table 1.** Overview of the experiment parameters

Experiment	Layer with Dynamic StO_2_	Blood tHb (μM)		μ_s_′ at 692 nm (cm^−1^)	SLT (mm)	Tested Devices
		Deep Layer	Superficial Layer			
#1	Deep	26	30	11.0	10 14 18	Masimo O3
#2	Deep	75	65	9.8	6 12 16	Masimo O3

#3	Deep	36	40	9.4	6 10 14 18	Masimo O3, INVOS 7100
#4	Superficial	60	65	9.0	6 10 14	Masimo O3, INVOS 7100

**Deep-Layer deoxygenation cycles**. Three experimental sessions were performed at total hemoglobin (tHb) concentrations of 26, 36, and 75 μM to cover a range of normal cerebral blood volumes. For each tHb concentration, we assessed different ranges of SLTs to determine the depth sensitivity of the NIRS devices (Table 1).

**Superficial-Layer deoxygenation cycles**. For the superficial layer (outer container) the tHb concentration was 65 μM (Table 1). For re-oxygenation two thin tubes were used to achieve a uniform O_2_ distribution throughout the larger surface area of the outer container.

**Mid-Level oxygenation stabilization**. To assess the influence of the superficial layers on the performance of the oximeters in more realistic physiological conditions, we kept StO_2_ at ∼75% in the inner container representing the brain. This corresponds to normal values [27]. For this purpose, we adjusted a steady O_2_ infusion to balance the yeast-driven.

## 3. Results

### 3.1. Optical stability of the phantom

Properly evaluating NIRS devices requires stable phantom properties. The phantom proved to indeed be stable for a long time in most of the experiments. The automated heating system consistently kept the temperature between 36-38°C without using excessive heat. We maintained a physiological pH ≈ 7.3-7.4 by adding small amounts of natrium bicarbonate (0.1-0.2 pH units) every two to three deoxygenation cycles. The optical properties were stable throughout the testing: μ_a_ remained unchanged during 6 h while μ_s_′ decreased by 10% after 2 h. Due to the good stability, the evaluation of the NIRS instrument is valid.

### 3.2. Response of NIRS devices to deep-layer oxygenation

**Fig. 3.**
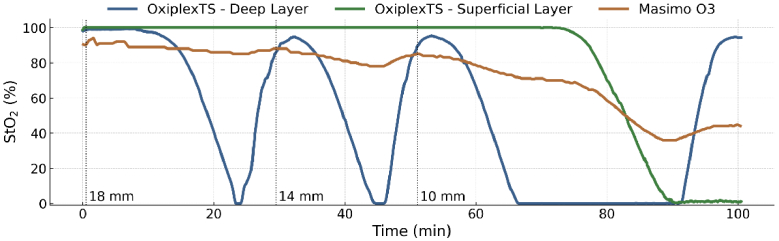
StO_2_ measurements with 26 μM hemoglobin (tHb) concentration. Blue and green lines show brain and superficial readings from the reference device (OxiplexTS). The orange shows Masimo O3 measurements from the multilayer window at three SLTs (18, 14, and 10 mm).

Figure 3 depicts a typical experimental time course: The OxiplexTS reference probes monitor changes in outer (green curve) and inner (blue curve) containers. There are several full-range oxygenation changes in the deep layer from nearly 100% to minimal StO_2_. The superficial layer’s thickness is actively decreased: the vertical dotted lines mark these changes. At minute 75, the superficial compartment is deoxygenated to assess the sensitivity of the NIRS device to superficial changes. The phantom demonstrates its dual capability to change its geometry and oxygenation patterns within both layers. In addition, from the range of the change in StO_2_, we can derive the sensitivity of an instrument to the deep layer.

**Fig. 4.**
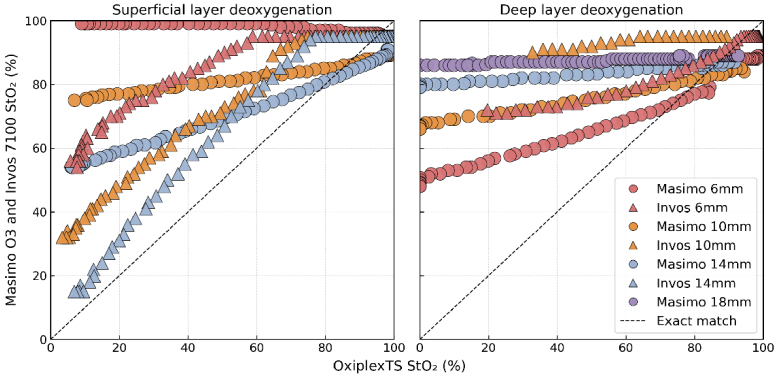
Agreement between Masimo O3, INVOS 7100, and the reference device (OxiplexTS) during deoxygenation cycles. Left: superficial layer (outer container) deoxygenation cycles with tHb = 65 μM. Right: brain layer (inner container) deoxygenation cycles with tHb = 36 μM. Dashed line indicates the line of identity.

**Fig. 5.**
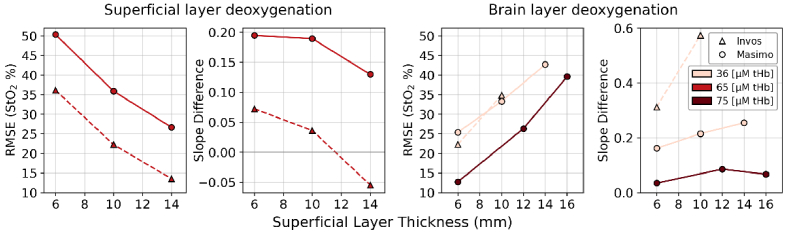
RMSE and slope difference of StO_2_ measurements versus changes in SLT for both superficial and deep deoxygenation. Calculated by comparing each device’s readings against the reference measurements.

The plots in Fig. 4 right compare Masimo O3, INVOS 7100 StO_2_ against the reference StO_2_ of the OxiplexTS for the deep layer at tHb = 36 μM. The Masimo O3 readings (circles) show a strong positive correlation with the deep-layer StO_2_ across all tested SLTs. However, these readings are consistently above the line of identity, which corresponds to a substantial bias. Masimo O3 showed progressively decreasing agreement with the brain reference as superficial thickness increased. The root mean square error (RMSE) values, shown in Fig. 5, were 25.4 at 6 mm, 33.3 at 10 mm, and 42.7 at 14 mm. The slope difference was used to quantify the similarity in the rates of change exhibited by the device and reference time series during the deoxygenation cycle. This metric is the arithmetic difference between two slopes: one from a linear line fitted to the device StO_2_ versus time, and the other from a linear line fitted to the corresponding reference StO_2_ versus time. The slope difference progressively increased from 0.16 (6 mm) to 0.21 (10 mm) and to 0.25 at 14 mm. A value closer to zero indicates that the overall rate of change in the device measurements more closely matches the rate of change in the reference measurements, demonstrating better agreement in tracking oxygenation dynamics. At the higher tHb concentration of 75 μM, the Masimo O3 again showed decreasing agreement with thickness increases (6 mm – 12 mm – 16 mm, RMSE: 12.7 - 26.3 - 39.6), but maintained better trend alignment (Slope difference: 0.03 - 0.09 - 0.07).

Masimo also demonstrated better accuracy at 6 mm (RMSE of 12.7 compared to 25.4) and improved overall trend alignment (slope difference close to zero) at tHb = 75 μM compared to tHb = 36 μM. However, the agreement decreased at a faster rate when superficial thickness increased during 75 μM tHb experiment. The INVOS 7100 device (triangles) was less sensitive to deep-layer dynamics. At the thinnest SLT of 6 mm, the red triangles showed a weak and scattered trend and the readings plateaued at 95%. The reason is that the INVOS clips values >95%. This masked physiological changes in deeper layers regardless of reference StO_2_ levels. The INVOS device showed low depth sensitivity for SLT > 10 mm, where the measurements consistently formed a straight line at the value of 95%. For this reason, the data are not displayed in the figures. INVOS exhibited reduced agreement from 6 mm to 10 mm while its RMSE increased from 21.3 to 33.8. The slope difference also showed a significant rise from 0.31 to 0.57 demonstrating a clear deviation from the reference signal trend.

**Fig. 6.**
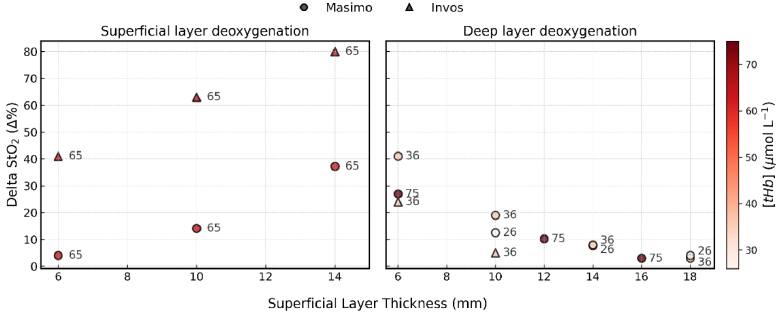
Overall dynamic range (ΔStO_2_) measured by Masimo O3 (circles) and INVOS 7100 (triangles) across deoxygenation cycles. Shown for varying SLTs and tHb concentrations (26, 36, 65, and 75 μM), and for deoxygenation in the superficial (left) and deep layer (right).

The overall dynamic range (ΔStO_2_) captured by each device across different experimental conditions is summarized in Fig. 6. The Masimo O3 sensor produced greater ΔStO_2_ ranges than the INVOS 7100 for the deep-layer. As SLT increases, the ΔStO_2_ ranges decrease for the Masimo O3. This means increased thicker layers decrease the sensitivity to deeper tissue, a common issue. The experimental data show that the INVOS consistently displays small ΔStO_2_ ranges, especially for SLTs of ≥10 mm. This shows limited depth sensitivity (Fig. 6).

### 3.3. Superficial-layer influence on NIRS readings

To evaluate the device sensitivity to superficial changes, we induced StO_2_ cycles in the superficial layer while maintaining stability in the deep layer. Tests were conducted at SLTs of 6 mm, 10 mm, and 14 mm. Figure 4 (left) showed how the devices react to changes in the superficial layer. The INVOS 7100 StO_2_ shows a considerable positive correlation with the reference StO_2_ of the superficial layer for all SLTs tested. The INVOS StO_2_ displayed the best match with superficial reference StO_2_ at 14 SLT. The strong correlation proved that the INVOS is mainly sensitive to superficial layers. At 6 mm, INVOS showed higher sensitivity to superficial tissue than Masimo: RMSE: 35.1 vs. 50.3. As SLT increased to 14 mm, contamination rose for both devices, but more substantially for INVOS, whose RMSE decreased by ∼64% compared to ∼47% for Masimo from the 6 mm baseline. At 14 mm, INVOS exhibited significantly greater sensitivity to the superficial layer (RMSE: 12.5 vs. 26.7). This finding was supported by the slope difference analysis, which showed INVOS slope difference relative to the reference, trending closer to zero (from 0.06 to -0.05 for INVOS, from 0.19 to 0.13 for Masimo, at 6 mm and 14 mm), indicating a closer match between the overall trend of the INVOS StO_2_ and the superficial reference StO_2_. The analysis of the superficial-layer experiments shown in Fig. 6 reveals that the INVOS 7100 showed higher ΔStO_2_ values than Masimo O3. This means the INVOS is more influenced by superficial layer changes, which matches the findings in Fig. 4. Masimo O3 is not influenced by a SLT = 6 mm. Its ΔStO_2_ is also influenced for SLT > 6 mm, but much less than INVOS.

### 3.4. Mid-level oxygenation stabilization

**Fig. 7.**
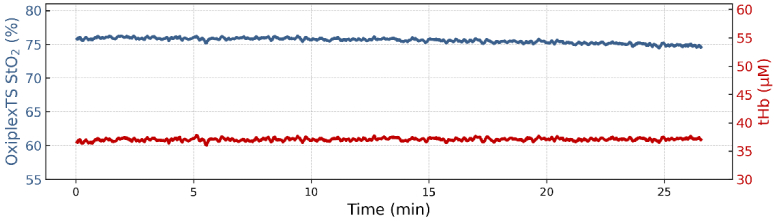
Time-course of the phantom stability at an intermediate oxygenation level. The plot shows tissue oxygen saturation (StO_2_, blue line, left axis) and total hemoglobin (tHb, red line, right axis). Measurements are taken with OxiplexTS device.

The phantom's ability to maintain a consistent, intermediate level of oxygenation (∼75% StO_2_) was tested within the deep compartment. For this experiment, a reduced yeast concentration compared to the dynamic tests was applied: we started with only 2 grams. Thus, the deoxygenation rate was slower. Once the desired StO_2_ was reached, to achieve a stable StO_2_, we started adding O_2_ gas gradually. We increased the O_2_ flow with a precise regulator until a stable equilibrium was reached. Stability occurs by precisely balancing yeast-driven deoxygenation and O_2_ infusion. The deep compartment's StO_2_ remained within 5% of the target level for ∼25 minutes before a small recalibration of the O_2_ infusion was necessary (Fig. 7). Thus, the phantom can stabilize at intermediate oxygenation levels, although this requires some fine-tuning.

## 4. Discussion

In this study, we proposed a multi-layer blood-lipid phantom to address limitations found in existing phantoms. Many previous models were not representative for the layered structure of the head or were lacking solid reference measurements. The present phantom consists of two dynamic liquid layers, that implement changes of oxygenation and tHb. The SLT is adjustable to simulate the brain surrounded by superficial tissues. Fixed solid layers represent other tissue parts and providing structural support. This design enables independent control over the optical properties of each layer, allowing the calibration of NIRS devices at a high level of accuracy and enabling more realistic modeling of physiological states [28]. One of the key features of this phantom is depicted in Fig. 3 is its ability to adjust the SLT. This enables to test NIRS devices for their depth sensitivity. Notably, when the deep layer is fully deoxygenated (0% StO_2_) and the superficial layer fully oxygenated (100% StO_2_), the StO_2_ of the tested device, yields the sensitivity of the device to the deep container. The equation: Sensitivity = 100%-StO_2_,_tested_device_ quantifies the contribution of the deep layer to the overall signal. In principle, the same is true for taking the ΔStO_2_ during deep layer deoxygenation, but this value directly quantifies the sensitivity in %. Quantification of the depth sensitivity is clinically relevant. The current study shows that the Masimo O3 has a higher depth sensitivity than the INVOS 7100, but at 10 mm SLT the sensitivity is only ∼20% (Masimo O3) and <10% for the INVOS 7100. 10 mm SLT plus the 0.9 mm most superficial and 1.9 mm intermediate solid window thickness are equal to a depth of the deep layer of 12.8 mm, which corresponds most closely to the depth of the brain inside the head. Thus, the phantom’s independent control over the oxygenation levels of layered compartments provides a direct quantitative measurement of the true physiological sampling depth of commercial NIRS devices. This is the most important achievement. In our study, the Masimo O3 demonstrated superior performance in tracking deep-layer StO_2_, as evidenced by its responsiveness in our multi-layer blood-lipid phantom model. This observation aligns with previous research emphasizing the importance of accurate deep tissue oxygenation measurements [29–32]. From physiology we can assume that there can be situations, where the StO_2_ of the brain differs from superficial tissue, e.g. due to the different arteries supplying the O_2_. From a clinical point of view, it is important that the StO_2_ of a NIRS device reflects the brain, because the brain is most sensitive to hypoxia, which may quickly lead to irreversible lesions and ensuing long-term disabilities.

Furthermore, we evaluated the feasibility of maintaining intermediate oxygenation levels (∼75% StO_2_) in the deep layer of our multi-layered phantom, reflecting typical steady-state cerebral oxygenation values observed in healthy adults [27]. This capability is particularly valuable for simulating physiologically relevant conditions during NIRS device validation. Previous studies have highlighted the need for real-time phantoms with tunable and stable optical properties, especially for dynamic or steady-state calibration under controlled environments [33–37]. Moreover, with an optimized dynamic interaction between deoxygenation processes and oxygen supply, we achieved stability within a 5% range, i.e., a level of stability that closely mimics real clinical conditions [38]. This level of control reaffirms the phantom technical potential to mirror specific clinical oxygenation scenarios accurately, something many previous designs could not replicate [39,40].

There are some limitations: Some mechanical aspects require refinement. The stepper-driven linkage platform positions the inner container with a small error of ≤ 0.5 mm over superficial-layer thicknesses (SLT) of 2 mm to 30 mm. Currently, friction in the printed guide tunnel leads to less smooth motion once the SLT exceeds 20 mm. Therefore, we have not used such larger thicknesses. Also, repeated cycles led to micro-droplet seepage at the front and lateral gaskets. Both issues stem from the tolerances of our 3D-printed PETG parts and would be solved by switching to machined metal rails, higher-resolution printing, or upgraded gasket compression. Implementing these changes would stabilize SLT adjustment and eliminate small leakage during long runs. However, these effects were small and thus do not affect our results.

Probes need to smaller than 65 mm in radius, otherwise they exceed our window. Still, most probes will fit into this type of window.

To keep the phantom stable at an intermediate StO_2_, this may require active fine-tuning. This could be solved by an automated oxygenation control, such as a closed-loop pO_2_ feedback system. This would extend the steady-state with higher stability without needing human intervention.

Furthermore, the present experiments merged the scalp and skull into a single perfused superficial layer separated from the brain layer by a solid phantom of only 1.9 mm, which contrasts with the in vivo situation where the scalp has a richer tHb than the skull. This may not align with the layered structure some device manufacturers expect. The use of interchangeable solid layers in our phantom design allows for the insertion of thicker bone-mimicking solid phantoms to match the optical model of a skull layer. Future work will explore this three-layer configuration.

Additionally, the capacity for simultaneous multi-device assessment is another point of consideration. The current design supports only one NIRS probe at a time. Previously published single-layer phantoms allowed multiple probes [4], which permits simultaneous device comparison within one compartment. Sudakou et al. [6] developed a dual-layer phantom that allows concurrent measurements in the deep layer. Future phantom iterations could address this limitation. Additional windows could be incorporated to allow measuring in parallel within individual superficial and deep compartments, following the approach by Sudakou et al. [6]; this would only lead to a moderate rise in size. Alternatively, a more substantial redesign could introduce two access points for assessing multiple layers simultaneously, for example, at the front and rear of the container. This development would provide an innovative way to comprehensively compare devices, a capability not yet available in existing multi-layer phantoms. Addressing these aspects will further enhance the phantom’s value in benchmarking NIRS technologies.

In summary, our multi-layer blood-lipid phantom is a relevant advance in simulating human tissue for NIRS validations. It not only solves problems of previous models, but also introduces new capabilities to facilitate improved assessments of commercial NIRS devices. The performance analysis of INVOS and Masimo O3 underscores the importance of evaluating aspects that are clinically relevant. This will facilitate developing NIRS devices that comply with these requirements.

## 5. Conclusion

We developed an adjustable dual-layer blood phantom that separately controls oxygenation in a deep and a superficial compartment. This is valuable for testing NIRS devices for accuracy and depth sensitivity, and will help improving non-invasive cerebral oximetry for clinical applications. The experimental results further demonstrate the relevance of these multi-layer phantoms in determining depth sensitivity. The Masimo O3 showed higher sensitivity to the deep layer and was less influenced by the superficial layer compared to the INVOS 7100. But at the superficial layer thickness of ≥ 10 mm, both instruments were mainly influenced by the superficial layer itself. It is important to reduce the influence of superficial tissue and increase the sensitivity to the deep tissue, e.g., for measuring the oxygenation of the brain. In the future, three-layer experiments involving scalp, skull (solid window), and brain compartments will be conducted, and commercial NIRS devices will be tested further.

## Data Availability

Data underlying the results presented in this paper are not publicly available at this time but they will be made available on a Zenodo repository at a later stage.
